# Handbook of immunohistochemistry and *in situ* hybridisation of human carcinomas: molecular genetics – lung and breast carcinomas

**DOI:** 10.1038/sj.bjc.6602863

**Published:** 2005-11-22

**Authors:** K C Gatter

**Affiliations:** 1 Nuffield Department of Clinical Laboratory Sciences, Room 4A123, John Radcliffe Hospital, Headington, Oxford, OX3 9DU, UK

These are the first two volumes of three forming a comprehensive overview of immunohistochemistry and molecular diagnosis of Human Carcinomas. Volume 1 deals with lung and breast cancer and volume 2 colorectal and prostate. Volume 3, which I have not seen, is apparently available and covers ovary, pancreas and liver.

Volumes 1 and 2 start with some general chapters on techniques and equipment that are of general relevance and then deal with their specific cancers in multiple short chapters. Most of these latter are reviews of immunohistochemical studies of a wide range of different markers such as mucins, laminin, VEGF and so on. They all tend to end in the same way that I was able to predict after reading two or three, that is, this marker may be useful but further studies are required. Almost nothing other than HER-2 in breast cancer was an established reagent. Most of the chapters are well written and illustrated and there is surprisingly little overlap between them, suggesting careful direction by the editor to his authors. Most chapters have references up to 2002 and a few even make 2003. This is pretty good for such a large book, but inevitably limits its usefulness in such a fast moving field. For example, there is currently a good deal of interest in quantitative mechanisms for assessing multitissue arrays, but the latest references here are 2002 and already out of date.

This is a good reference work and probably one of the best of its kind, but one must question its relevance today. Who is going to buy these for the office at £110 each (even though I accept this is good value for such a tome)? Who is going to go down to the library to consult them when more up to date information is available at a few keystrokes on the desktop computer (even now on the laptop on the fly)? Books are still valuable tools for diagnosis or methods but this is neither. You would not use this to diagnose breast cancer and you would not get enough detail to undertake a new technique from the outlines given. Preinternet such books were vital but are they now obsolete? The publishers obviously hope not but the sales will tell the true tale.

The preface to Volume 1 written by Dr Hayat is very interesting. He outlines the objectives of this book as discussion of the various procedures and techniques and of the role of molecular pathology in achieving correct diagnosis and therapy. The first the books handle quite well but the second is problematical. Clinical practice, he says, has lagged behind molecular genetics. One reason for this is that it needs to be handled by clinician – scientists or academic clinicians. There is a dearth of these because of poor pay. Amen to that we might all say but are we diagnosticians such dullards? Are we short-changing our patients because of our Luddite inability to implement new tests. Well on reading these two volumes, I could not see a test that works that we are not using generally in the UK in practice, and I imagine that holds generally in the developed world. When antibodies are needed we use them and when genetic testing is important as in acute leukaemia we implement it. I sometimes think we do a disservice when we preach on about how backward are our clinical colleagues. Most of the studies in these books, fun as they may have been to perform, have no currently recognisable diagnostic value and probably never will. We should not forget that most of our research is not useful; we just do not yet know how to identify in advance those few kernels of gold.

